# Genome-wide association and genomic prediction for a reproductive index summarizing fertility outcomes in U.S. Holsteins

**DOI:** 10.1093/g3journal/jkad043

**Published:** 2023-02-27

**Authors:** Christopher M Seabury, Johanna L Smith, Miranda L Wilson, Eric Bhattarai, Jose E P Santos, Ricardo C Chebel, Klibs N Galvão, Gustavo M Schuenemann, Rodrigo C Bicalho, Rob O Gilbert, Sandra L Rodriguez-Zas, Guilherme Rosa, William W Thatcher, Pablo J Pinedo

**Affiliations:** Department of Veterinary Pathobiology, College of Veterinary Medicine, Texas A&M University, College Station, TX 77843, USA; Department of Veterinary Pathobiology, College of Veterinary Medicine, Texas A&M University, College Station, TX 77843, USA; Department of Veterinary Pathobiology, College of Veterinary Medicine, Texas A&M University, College Station, TX 77843, USA; Department of Veterinary Pathobiology, College of Veterinary Medicine, Texas A&M University, College Station, TX 77843, USA; Department of Animal Sciences, University of Florida, Gainesville, FL 32611, USA; Department of Large Animal Clinical Sciences, College of Veterinary Medicine, University of Florida, Gainesville, FL 32611, USA; Department of Large Animal Clinical Sciences, College of Veterinary Medicine, University of Florida, Gainesville, FL 32611, USA; Department of Veterinary Preventative Medicine, College of Veterinary Medicine, The Ohio State University, Columbus, OH 43210, USA; Department of Population Medicine and Diagnostic Sciences, College of Veterinary Medicine, Cornell University, Ithaca, NY 14850, USA; Department of Clinical Sciences, School of Veterinary Medicine, Ross University, St. Kitts, West Indies, KN; Department of Animal Sciences, University of Illinois at Urbana-Champaign, Urbana-Champaign, IL 61801, USA; Department of Animal Sciences, University of Wisconsin, Madison, WI 53706, USA; Department of Animal Sciences, University of Florida, Gainesville, FL 32611, USA; Department of Animal Sciences, Colorado State University, Fort Collins, CO 80521, USA

**Keywords:** GWAA, QTL, Holstein, fertility

## Abstract

Subfertility represents one major challenge to enhancing dairy production and efficiency. Herein, we use a reproductive index (RI) expressing the predicted probability of pregnancy following artificial insemination (AI) with Illumina 778K genotypes to perform single and multi-locus genome-wide association analyses (GWAA) on 2,448 geographically diverse U.S. Holstein cows and produce genomic heritability estimates. Moreover, we use genomic best linear unbiased prediction (GBLUP) to investigate the potential utility of the RI by performing genomic predictions with cross validation. Notably, genomic heritability estimates for the U.S. Holstein RI were moderate (*h*^2^ = 0.1654 ± 0.0317–0.2550 ± 0.0348), while single and multi-locus GWAA revealed overlapping quantitative trait loci (QTL) on BTA6 and BTA29, including the known QTL for the daughter pregnancy rate (DPR) and cow conception rate (CCR). Multi-locus GWAA revealed seven additional QTL, including one on BTA7 (60 Mb) which is adjacent to a known heifer conception rate (HCR) QTL (59 Mb). Positional candidate genes for the detected QTL included male and female fertility loci (i.e. spermatogenesis and oogenesis), meiotic and mitotic regulators, and genes associated with immune response, milk yield, enhanced pregnancy rates, and the reproductive longevity pathway. Based on the proportion of the phenotypic variance explained (PVE), all detected QTL (*n* = 13; *P* ≤ 5e − 05) were estimated to have moderate (1.0% < PVE ≤ 2.0%) or small effects (PVE ≤ 1.0%) on the predicted probability of pregnancy. Genomic prediction using GBLUP with cross validation (*k* = 3) produced mean predictive abilities (0.1692–0.2301) and mean genomic prediction accuracies (0.4119–0.4557) that were similar to bovine health and production traits previously investigated.

## Introduction

One persistent challenge to maximizing U.S. dairy production while also enhancing sustainability via genomic selection is subfertility among Holstein cows and heifers, with low milk production due to impaired fertility being one of the most frequent reasons for culling and a major obstacle to increasing dairy profitability (Beerda *et al*. 2008; Pinedo and de Vries 2010; Kiser *et al*. 2019). In response to concerns about low fertility among U.S. dairy herds, both pregnancy rates and conception rates have historically been recorded, and in 2003, a national genetic evaluation program for the daughter pregnancy rate (DPR) was also introduced, where records on days open were transformed to pregnancy rates using a linear function (VanRaden *et al*. 2004). However, because the DPR reflects the percentage of a bull's daughters that become pregnant within a 21-day period without accounting for the total services required or whether the females were even exposed to a breeding (AIPL 2005; Kiser *et al*. 2019), additional measures of fertility have also been investigated for incorporation into modern genetic evaluations and selection indices (Holstein Association USA 2014; Kiser *et al*. 2019). Examples of such measures include cow or heifer conception rates (CCR, HCR), which were utilized in genetic evaluations beginning in 2010 (AIPL 2010). Modern evaluations routinely performed for female fertility traits in U.S. Holsteins currently include HCR, CCR, DPR, and calving to first insemination (CFI), whereas those routinely employed by Interbull include maiden heifer's ability to conceive (HC), lactating cow's ability to recycle after calving (CR), lactating cow's ability to conceive expressed as rate trait (C1), lactating cow's ability to conceive expressed as an interval trait (C2), and lactating cow's measurements of interval traits for calving–conception (IT) (Interbull 2008; CDCB 2013). Notably, most of the Interbull traits have some degree of overlap with those routinely evaluated for U.S. Holsteins, with Interbull also providing some flexibility via alternative measures that can be submitted for three of the five routinely evaluated traits (Interbull 2008; CDCB 2013). Relevant to U.S. Holsteins, while generation intervals and HCR have both improved since 2010 (Garcia-Ruiz *et al*. 2016; CDCB 2017), subfertility still remains a primary obstacle to maximizing dairy production, sustainability, and profitability (Garcia-Ruiz *et al*. 2016; CDCB 2017; Kiser *et al*. 2019; Pinedo *et al*. 2020). Therefore, a need exists to explore new measures of Holstein fertility and to assess how the inclusion of those new measures may potentially augment modern genetic evaluation programs and selection indices.

Herein, we use a reproductive index (RI) expressing the predicted probability of a pregnancy previously developed for comparison and ranking of Holstein cow fertility across diverse U.S. geographic regions (Lopes *et al*. 2020; Pinedo *et al*. 2020) to conduct genome-wide association analysis (GWAA) with marker-based heritability estimates (Kang *et al*. 2010; Segura *et al*. 2012; Vihjálmsson 2012). Additionally, we also use genomic best linear unbiased prediction (GBLUP) with *k*-fold cross validation (Taylor 2014; Seabury *et al*. 2020) to explore the potential for using the RI in existing or future genomic selection initiatives aimed at improving Holstein cow fertility. The results of this study are expected to potentially enhance future genetic evaluation programs while also elucidating single nucleotide polymorphisms (SNPs) and positional candidate genes associated with holistic differences in Holstein cow fertility.

## Materials and methods

### Holstein study population, RI development, DNA isolation, and genotyping

From 2012 to 2014, a total of 11,733 Holstein cows calving on 16 dairy farms located in four U.S. regions (northeast, *n* = 4 herds; midwest, *n* = 6 herds; southeast, *n* = 1 herd; southwest, *n* = 5 herds) were enrolled in a fertility study beginning at parturition and subsequently monitored for both health and reproductive events by members of the research team using standardized protocols (Lopes *et al*. 2020; Pinedo *et al*. 2020). Enrollment and cow monitoring were conducted under typical production practices, and did not require euthanasia. Monitoring for disease, body condition score, and lameness was performed on weekly visits to the study farms, as previously described (Lopes *et al*. 2020; Pinedo *et al*. 2020). Disease events recorded included retained fetal membranes (RFM), metritis (MET), clinical mastitis (MAST), displaced abomasum (DA), lameness (LS), clinical endometritis (CE), respiratory disease (RD), dystocia (DYS), and subclinical ketosis (SCK), with records on monthly milk tests, days in milk (DIM), and milk yield to 305 days also obtained, as previously described (Lopes *et al*. 2020; Pinedo *et al*. 2020).

Using the phenotypic information acquired from individual cows, a Holstein RI was previously developed and described, thus representing the predicted probability that a cow will become pregnant, as a function of explanatory variables and interactions identified in a logistic regression model (*P* < 0.05) (Lopes *et al*. 2020; Pinedo *et al*. 2020). Specifically, the final RI model (RI2) included significant fixed effects and interactions as explanatory variables influencing Holstein pregnancy as follows: U.S. region, parity number, body condition score at 40 DIM, RFM, MET, resumption of ovulation by 50 DIM, SCK, CE, MAST, milk yield at the first milk test after calving, the interaction effect of resumption of ovulation by 50 DIM × region, the interaction of MAST × region, and the interaction of milk yield at the first milk test after calving × parity number (Lopes *et al*. 2020; Pinedo *et al*. 2020). Therefore, the Holstein RI utilized in this study (RI2) is a composite variable originating from the probability equation of the logistic regression model, thus representing a continuous variable ranging from zero to one and is directly related to the probability of pregnancy, as previously described (Lopes *et al*. 2020; Pinedo *et al*. 2020).

Holstein cows were selected for genotyping on the Illumina 778K assay (Illumina, San Diego, CA) by defining two groups within each farm and calving season as follows: high-fertility individuals (diagnosed pregnant at 60 days after the first postpartum artificial insemination ) and among the highest 15% RI (*n* = 850) and low-fertility individuals (diagnosed not pregnant at 60 days after two postpartum AI attempts) and among the lowest 7.25% RI (*n* = 1,750), as recently described (Lopes *et al*. 2020; Pinedo *et al*. 2020), thus sampling from the tails of the RI distribution (Li *et al*. 2019). Moreover, because cows were selected from the tails of the RI distribution within each farm and calving season, the distribution of high and low RI phenotypes across farms is overlapping and without truncation (See Supplementary Fig. 1). Summary statistics for milk yield (up to 90 days of lactation, MAVG90kg) in high-fertility and low-fertility individuals are described in Additional File 4 (File S4). After selection, genomic DNA was isolated from bovine blood using the Promega Wizard Genomic DNA Purification Kit (Promega, Madison, WI) according to the manufacturer's recommendations. Genomic DNAs were quantified and evaluated for purity (260/280 ratio) via NanoDrop (ThermoFisher Scientific, Waltham, MA). Collectively, 2,505 of the selected cows produced high-quality genomic DNA (96.3%) and were submitted to GeneSeek Neogen (GeneSeek, Lincoln, NE) for Illumina Bovine 778K genotyping.

### GWAA and genomic prediction with cross validation

Prior to GWAA's and genomic predictions with cross validation, sample and genotype quality control analyses and filtering were performed as follows: sample call rate (<90% excluded), and thereafter, SNP filtering by call rate (>15% missing excluded), MAF (<0.01 excluded), and Hardy–Weinberg equilibrium (excludes SNPs with HWE *P*-value < 1e − 25), which yielded 2,448 Holstein cows and 628,526 SNPs for analysis. For GWAA's and genomic predictions with cross validation, the Holstein RI (also referred to as IndexP60) was used as the dependent variable (Lopes *et al*. 2020; Pinedo *et al*. 2020). All Holstein GWAAs were performed using a mixed linear model with variance component estimates, as implemented in efficient mixed-model association expedited (EMMAX), and executed in SVS v8.8.3 (Golden Helix), where all genotypes are recoded (i.e. 0, 1, or 2) based on the incidence of the minor allele (Kang *et al*. 2010; Segura *et al*. 2012; Smith *et al*. 2019; Seabury *et al*. 2020; Seabury et al. 2017). The general mixed model can be specified as follows: y=Xβ+Zu+ϵ, where *y* represents a *n* × 1 vector of Holstein RI phenotypes, *X* is a *n* × *f* incidence matrix of fixed effects, *β* is a *f* × 1 vector representing the coefficients of the fixed effects, *u* represents the random effect of cow, and *Z* is a *n* × *t* incidence matrix relating the random effects to the Holstein RI phenotypes of interest (Kang *et al*. 2010; Segura *et al*. 2012; Seabury *et al*. 2020; Seabury *et al*. 2017; Smith *et al*. 2019). Herein, we must assume that $\mathrm{Var}(u)=\sigma_{g}^{2}K$ and $\mathrm{Var}(\epsilon )=\sigma_{e}^{2}I$, such that $\mathrm{Var}(y)=\sigma_{g}^{2}ZKZ^{\prime }+\sigma_{e}^{2}I$, but in the present study, *Z* represents the identity matrix *I*, and *K* represents a relationship matrix of all Holstein samples. To solve the mixed model equation, we must estimate the variance components (i.e. $\sigma_{g}^{2}$ and $\sigma_{e}^{2}$) as previously described (Kang *et al*. 2010; Segura *et al*. 2012; Smith *et al*. 2019; Seabury *et al*. 2020; Seabury *et al.* 2017). Briefly, variance components were estimated using the REML-based (restricted maximum likelihood) EMMA approach (Kang *et al*. 2010), with stratification accounted for and controlled using a genomic relationship matrix (GRM; *G*) (VanRaden 2008), as computed from the Holstein genotypes. All GRM heritability estimates ($\mathrm{Genomic}\;h^{2}=\sigma_{g}^{2}/(\sigma_{g}^{2}+\sigma_{e}^{2})$ for the Holstein RI were produced as previously described (Kang *et al*. 2010; Segura *et al*. 2012; Smith *et al*. 2019; Seabury *et al*. 2020; Seabury et al. 2017). In addition to the original single-locus approach described and implemented within EMMAX (Kang *et al*. 2010), we also performed a multi-locus extension of the EMMAX procedure which involves mixed-model regression with forward inclusion and backward elimination of genotypic markers used as fixed effect covariates (“cofactors”) (Segura *et al*. 2012; Vihjálmsson 2012). Briefly, the multi-locus approach begins with an initial model that includes only the intercept and the specified covariates as fixed effects. Thereafter, the EMMAX procedure was performed across all Illumina Bovine 778K HD SNPs individually, and the most significant SNP was then added to the model as a new fixed effect covariate (“cofactor”), thereby creating a new model, with this forward selection procedure repeated for 10 steps (Kang *et al*. 2010; Segura *et al*. 2012; Vihjálmsson 2012). Likewise, 10 steps of backward elimination were also performed, and each SNP included in the current model as a fixed effect covariate (cofactor) was temporarily removed, the EMMAX procedure was performed, and the least significant SNP included in the model was removed, until only one SNP remained (Kang *et al*. 2010; Segura *et al*. 2012; Vihjálmsson 2012). Variance components (i.e. $\sigma_{g}^{2}$ and $\sigma_{e}^{2}$) were also estimated in a stepwise manner, as previously described, and genomic heritability estimates were produced (Kang *et al*. 2010; Segura *et al*. 2012; Smith *et al*. 2019; Seabury *et al*. 2020; Seabury et al. 2017). The model optimality criteria used to evaluate forward and backward steps included the Bayesian information criterion (BIC) as follows: BIC = −2*l*_*F*_ + *p* × log(*n*), where *l*_*F*_ is the full-model log-likelihood, *p* is the number of model parameters (i.e. one for the intercept, one for *δ*, one for each genotypic marker covariate (cofactor) used in the particular multi-locus model, and one for each additional fixed effect covariate used in all of the models), where *n* is the sample size; Extended Bayesian Information Criterion (EBIC) as follows: $\mathrm{EBIC}=\mathrm{BIC}+2\log\;((\begin{array}{c} n\\ \;p-q \end{array}))=\mathrm{BIC}+2(\sum_{i=n-(\;p-q)+1}^{n}\;\log\;(i)+\sum_{i=1}^{\;p-q}\;\log(i))$, where *q* is the initial number of model parameters (i.e. one for the intercept, one for *δ*, and one for each additional covariate used in all of the models) and with $(\begin{array}{c} n\\ \;p-q \end{array})$ being the total number of models possible using *p* − *q* genotypic marker covariates (cofactors) under the assumption that these will only be selected from the best *n* markers; the related Modified Bayesian Information Criterion (MBIC) as follows: $\mathrm{MBIC}=\mathrm{BIC}+2p\times \log\left(\frac{m}{2.2}-1\right)$, with *m* representing the total number of markers being tested in the current multilocus step; Bonferroni criterion as: for forward selection models only, and an optimal model is defined as the model with the most genotypic marker covariates (cofactors) for which the best *P*-value obtained from the preceding EMMAX scan was below the Bonferroni threshold; Multiple Bonferroni Criterion as follows: an optimal model is the model with the most genotypic marker covariates (cofactors) whereby all of which have individual *P*-values below the Bonferroni threshold, with the threshold used being 1/(20*m*) and with *m* being the total number of markers tested in the current multi-locus step; and Multiple Posterior Probability of Association as follows: an optimal model is the model with the most genotypic marker covariates (cofactors) where all have posterior probabilities of association ≥ 0.50. Posterior probabilities of association are based on Bayesian priors pr of 1/*m* for every marker (and for every step), with *m* being the total number of markers being tested in the current multi-locus step and where the Bayesian factor for marker *k* is as follows: $\mathrm{bf}=\exp(\frac{n\log(\frac{\mathrm{mrs}s_{h0}}{\mathrm{mrs}s_{k}})-\log\;(n)}{2})$, with mrss_*h*0_ and mrss_*k*_ being the values of the Mahalanobis Residual Sum of Squares (RSS) for the base model and for testing with the marker *k*, respectively. Posterior odds and posterior probability were calculated as follows: $\mathrm{po}=\mathrm{bf}\frac{\mathrm{pr}}{1-\mathrm{pr}}$ and $\mathrm{pp}=\frac{\mathrm{po}}{1+\mathrm{po}}$. In all EMMAX GWAAs, we specified farm and season of calving as fixed-effect covariates. All QTL were defined by ≥ 3 SNP loci (i.e. a lead SNP plus at least two additional supporting SNP within 1 Mb) which also met a nominal significance threshold for polygenic traits (*P* ≤ 5e − 05) (Wellcome Trust Case Control Consortium 2007).

For all genomic predictions with *k*-fold cross validation (*k* = 3), we used GBLUP as previously described and implemented in SVS v8.8.3 (Golden Helix), where we again estimate the variance components using the REML-based EMMA technique with a GRM (*G*) (VanRaden 2008; Kang *et al*. 2010; Taylor 2014; Seabury *et al*. 2020). For the U.S. Holstein GBLUP analyses, the general mixed model equation can be specified as: $y=X_{f}B_{f}+u+\epsilon$, across *n* U.S. Holstein cows where fixed effects specified as *B*_*f*_ include the intercept and any additional covariates (i.e. farm and season of calving) but also assume that $\mathrm{Var}(\epsilon )=\sigma_{e}^{2}I$, as described above, and that the random effects *u* are the additive genetic merits [i.e. genomically estimated breeding values (GEBVs)] for these U.S. Holstein cows, which are produced from *m* genotypic markers as *u* = *Mα*, where *M* is a *n* × *m* matrix and *α* is a vector where *α*_*k*_ is the allele substitution effect (ASE) for marker *k*. For this study, we used overall normalization for matrix *M*, as implemented in SVS v8.8.3 (Golden Helix), and explored solutions with and without fixed effect covariates (i.e. farm, season of calving), to produce GEBVs for all Holstein cows as well as estimates of ASE for all SNPs (VanRaden 2008; Kang *et al*. 2010; Taylor 2014; Seabury *et al*. 2020). For cross validation, considering that all Holstein RI training set samples precede the validation set, we define *Z* = [*I*|0], where the width and height of *I* are given as *n*_*t*_, the width of the zero matrix is given as *n*_*v*_, and the height of the zero matrix is *n*_*t*_. Therefore, we can partition *u*, *X*_*f*_, and *y* by their origin (i.e. training versus validation set) as $u=\left[\begin{array}{c} u_{t}\\ u_{v} \end{array}\right]$, $X_{f}=\left[\begin{array}{c} X_{\;ft}\\ X_{\;fv} \end{array}\right]$, $y=\left[\begin{array}{c} y_{t}\\ y_{v} \end{array}\right]$ and compute a GRM using all Holstein samples for use with the EMMA technique (VanRaden 2008; Kang *et al*. 2010; Seabury *et al*. 2020); to implement a mixed model for the training set as follows: $y_{t}=X_{ft}B_{f}+Zu+\epsilon_{t}$, where $\mathrm{Var}(u)=\sigma_{G}^{2}G$ and $\mathrm{Var}(Zu)=\sigma_{G}^{2}ZGZ^{\prime }$. Herein, we predict the Holstein validation set RI phenotypes as follows: ${\hat{y}}_{v}=X_{fv}{\hat{B}}_{f}+{\hat{u}}_{v}$, from the intercept and any validation covariates *X*_*fv*_ as well as the predicted values of $\;{\hat{u}}_{v}$. Thus, our cross validations produce the opportunity to evaluate both the predicted Holstein RI phenotypes and the Holstein GEBVs in relation to the known Holstein RI phenotypes (Seabury *et al*. 2020). Additional formulae and supporting documentation are available at https://doc.goldenhelix.com/SVS/latest/svsmanual/mixedModelMethods/overview.html#gblupproblemstmt. For the GBLUP predicted Holstein RI phenotypes, we report relevant summary statistics from the *k*-fold (*k* = 3) cross validations (*n* = 10 iterations) computed in SVS v8.8.3 (Golden Helix) as follows: Pearson's Product–Moment Correlation Coefficient as $r_{y,\hat{y}}=\frac{\sum_{i=1}^{n}\left(y_{i}-\bar{y}\right)\left({\hat{y}}_{i}-\hat{\bar{y}}\right)}{\left(n-1\right)s_{y}s_{\hat{y}}}$ where *s*_*y*_ and $s_{\hat{y}}$ are the standard deviations; Residual Sum of Squares as $\mathrm{RSS}=\sum_{i=1}^{n}\;(y_{i}-\hat{y}{)}^{2}$; Total Sum of Squares as $\mathrm{TSS}=\sum_{i=1}^{n}\;{\left(y_{i}-\bar{y}\right)}^{2}$; Coefficient of Determination as $R^{2}=1-\frac{\mathrm{RSS}}{\mathrm{TSS}}$; Root Mean Square Error as $\mathrm{RMSE}=\sqrt{\frac{\mathrm{RSS}}{n}}$; and Mean Absolute Error as $\mathrm{MAE}=\frac{1}{n}\sum_{i=1}^{n}\;|y_{i}-{\hat{y}}_{i}|$. Additionally, we also report predictive ability, as defined by the mean Pearson's Product–Moment Correlation Coefficient between the Holstein GEBVs and the known Holstein RI phenotypes for all validation sets (*k* = 3; *n* = 10 iterations) (Lopes *et al*. 2020), and genomic prediction accuracy, as defined by the mean Pearson's Product–Moment Correlation Coefficient between the Holstein GEBVs and the known Holstein RI phenotypes for all validation sets (*k* = 3; *n* = 10 iterations), divided by the genomic heritability estimate (Bolormaa *et al*. 2013). Genomic inflation factors were estimated in SVS v8.8.3 (Golden Helix) as follows: Pseudo-Lambda = log10(median observed *P*-value)/log10(median expected *P*-value).

### Randomizing and blinding

All GBLUP *k*-fold (*k* = 3) cross validations were performed using automated random sampling to define the validation set (i.e. to predict on) and the training set, for the specified value of *k*, with all validation set phenotypes set to missing in each fold.

## Results and discussion

### Heritability estimates for a U.S. Holstein RI

We utilized two related approaches implemented by EMMAX (Kang *et al*. 2010; Segura *et al*. 2012; Vihjálmsson 2012) to generate marker-based heritability estimates for the U.S. Holstein RI (Lopes *et al*. 2020; Pinedo *et al*. 2020). Specifically, genomic relationship matrices (GRM) (VanRaden 2008) scaled via Gower's centering approach were used to compare genomic heritability estimates ($h^{2}=\sigma_{g}^{2}/(\sigma_{g}^{2}+\sigma_{e}^{2})$) produced for both single-locus and multi-locus mixed linear models (Kang *et al*. 2010; Segura *et al*. 2012; Vihjálmsson 2012). All EMMAX approaches to GWAA also produced moderate heritability estimates with small standard errors (i.e. *h*^2^ = 0.1654 ± 0.0317– 0.1826 ± 0.0326), regardless of model optimality criteria (Table 1). Higher genomic heritability estimates were produced by both EMMAX (*h*^2^ = 0.2539 ± 0.0347) and GBLUP (*h*^2^ = 0.2550 ± 0.0348) when no fixed-effect covariates (i.e. farm,season of calving) were included in the models. However, the proportion of variance explained by the fixed-effect covariates (0.1193) suggests overestimation of these genomic heritability estimates, which is somewhat unsurprising considering that Holstein cows were selected for genotyping by defining two groups (i.e. high fertility and low fertility) within each farm and calving season (see methods). Nevertheless, moderate heritability estimates for the U.S. Holstein RI produced here (*h*^2^ = 0.1654 ± 0.0317– 0.1826 ± 0.0326) support the potential for positive economic and production gains resulting from the implementation of genomic selection; particularly since low heritability estimates have routinely been produced for most dairy fertility traits worldwide (Garcia-Ruiz *et al*. 2016; Kiser *et al*. 2019).

**Table 1.** EMMAX variance component analysis with genomic heritability estimates.

EMMAX*^a^* model (RI)	Multilocus*^b^* step type	Model optimality*^c^* criteria	GRM*^d^ h*^2^	Variance of *h*^2^	SE of *h*^2^	EMMA Vg	EMMA Ve
SL-Initial	NA	NA	0.1654	0.0010	0.0317	0.0021	0.0106
AF-Step 01 ML	FS		0.1774	0.0010	0.0321	0.0022	0.0102
AF-Step 02 ML	FS		0.1803	0.0010	0.0322	0.0022	0.0100
AF-Step 03 ML	FS		0.1833	0.0011	0.0324	0.0022	0.0098
AF-Step 04 ML	FS	MPPA	0.1780	0.0010	0.0323	0.0021	0.0097
AF-Step 05 ML	FS		0.1880	0.0011	0.0329	0.0022	0.0095
AF-Step 06 ML	FS	Bonf	0.1754	0.0011	0.0325	0.0020	0.0094
AF-Step 07 ML	FS		0.1770	0.0010	0.0324	0.0020	0.0093
AF-Step 08 ML	FS		0.1786	0.0011	0.0325	0.0020	0.0092
AF-Step 09 ML	FS		0.1636	0.0010	0.0321	0.0018	0.0092
AF-Step 10 ML	FS		0.1468	0.0010	0.0314	0.0016	0.0093
AF-Step 11 ML	BE		0.1455	0.0010	0.0313	0.0016	0.0094
AF-Step 12 ML	BE		0.1532	0.0010	0.0317	0.0017	0.0094
AF-Step 13 ML	BE		0.1770	0.0011	0.0324	0.0020	0.0093
AF-Step 14 ML	BE		0.1770	0.0011	0.0325	0.0020	0.0093
AF-Step 15 ML	BE	MBonf, EBIC	0.1826	0.0011	0.0326	0.0021	0.0094
AF-Step 16 ML	BE	MBIC	0.1724	0.0010	0.0324	0.0020	0.0096
AF-Step 17 ML	BE		0.1765	0.0010	0.0322	0.0021	0.0098
AF-Step 18 ML	BE		0.1736	0.0010	0.0320	0.0021	0.0100
AF-Step 19 ML	BE		0.1840	0.0011	0.0327	0.0023	0.0102

a SL, single-locus EMMAX (SL) initial model; AF-Step, after step; ML, multi-locus EMMAX.

b NA, not applicable; FS, forward selection; BE, backward elimination.

c MPPA, multiple posterior probability of association; Bonf, Bonferroni; MBonf, multiple Bonferroni; EBIC, extended Bayesian information criterion; MBIC, modified Bayesian information criterion.

d EMMAX genomic heritability estimates (Kang et al. 2010; Segura et al. 2012; Vihjálmsson 2012).

### GWAA for a U.S. Holstein RI

**Fig. 1.**
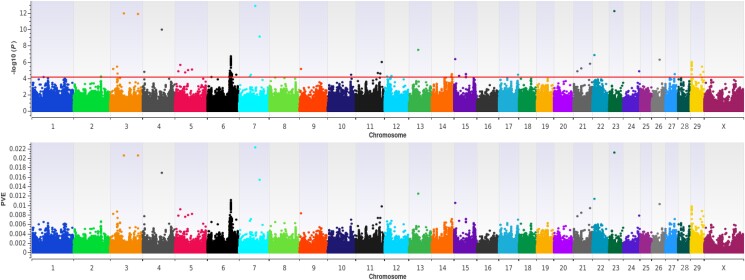
QTL detected for a U.S. Holstein RI by EMMAX (single locus). The dual-panel Manhattan plot depicts −log10 *P*-values and the proportion of phenotypic variance explained (PVE) by bovine marker effects on the *y*-axis and the genomic position of all SNPs on the *x*-axis (UMD3.1.1). Lead and supporting SNPs for QTL passing a nominal significance threshold for polygenic traits (*P* ≤ 5e−05; −log_10_*P*-values ≥ 4.30) (Wellcome Trust Case Control Consortium 2007) are represented at or above the red line for *n* = 2,448 U.S. Holstein cows. A summary of detected QTL, including lead and supporting SNPs passing the nominal significance threshold, is presented in Table 2 and Additional File 1 (File S1) in DRYAD:10.5061/dryad.0gb5mkm04.

The results of our Illumina 778K single-marker analysis (EMMAX) (Kang *et al*. 2010) are shown in Fig. 1 and in Additional File 1 (File S1), with detailed summary data for QTL detected by EMMAX described in Table 2. All Holstein QTL were defined and localized by lead SNPs (i.e. the most strongly associated SNP within a QTL region) which met a nominal significance threshold for polygenic traits (*P* ≤ 5e − 05) (Wellcome Trust Case Control Consortium 2007). Notably, the largest-effect QTL detected for the U.S. Holstein RI (PVE = 0.0110 or 1.1%) was located on BTA6 (89 Mb) and included positional candidate genes (i.e. *SLC4A4* and *GC*) previously associated with Holstein milk and protein yields, somatic cell score or clinical mastitis, DPR, and CCR (Table 2) (Sahana *et al*. 2014; Jiang *et al*. 2019). Additional QTL signals detected on BTA29 (4 Mb) and BTA6 (84 Mb, 85 Mb, 88 Mb, and 93 Mb) revealed three additional positional candidate genes previously associated with high or low fertility in beef heifers (*DCK*) (Killeen *et al*. 2014), the regulation of mitosis and meiosis (*CENPC*) in humans and *Drosophila* (i.e. spermatogenesis) (Saitoh *et al*. 1992; Przewloka *et al*. 2011; Kwend *et al*. 2016), and germ cell development (*SDAD1*) in planarian, humans, and fish (i.e. spermatogenesis, oogenesis; see Table 2) (Fox *et al*. 2005; Gautier *et al*. 2011; Iyer *et al*. 2016). All QTL detected for the U.S. Holstein RI were estimated to have moderate (PVE ≥ 1.0% but < 2.0%) or small effects (PVE ≤ 1.0%) on the probability of a pregnancy following artificial insemination (see Table 2); with no evidence of spurious associations via population substructure, as evidenced by the estimated genomic inflation factor (0.9935).

**Table 2.** Holstein QTL detected by single-locus EMMAX analysis for a reproductive index (RI).

QTL*^a^* Chr_Mb	EMMAX −log_10_*P*-value	Regression beta	PVE	Supporting SNPs*^b^*	Positional candidate genes	Lead SNP location	Scientific precedence (reference); organism; trait
***6_89***	6.6780	−0.0188	0.0110	36	*SLC4A4*, *LOC782958*, *GC*	Intergenic	(Sahana et al. 2014; Jiang et al. 2019); cattle; milk and protein yields, mastitis, DPR, CCR
***29_04***	5.9362	−0.1736	0.0097	10	*LOC101907665*, *LOC104976210*	Intergenic	NA
***6_88***	5.9251	−0.0183	0.0097	2	*DCK*, *SLC4A4*, *GC*	Intergenic	(Killeen et al. 2014; Sahana et al. 2014; Jiang et al. 2019); cattle; milk and protein yields, mastitis, DPR, CCR high or low fertility
***6_85***	4.9231	0.0164	0.0079	3	*LOC100139136*, *LOC101908113*, *CENPC*	Intergenic	(Saitoh et al. 1992; Przewloka et al. 2011; Kwend et al. 2016); humans; *Drosophila*; regulation of mitosis and meiosis; spermatogenesis
***6_84***	4.6166	0.0161	0.0073	4	*LOC100139136*, *LOC101908113*, *CENPC*	Intergenic	(Saitoh et al. 1992; Przewloka et al. 2011; Kwend et al. 2016); humans; Drosophila; regulation of mitosis and meiosis; spermatogenesis
***6_93***	4.4942	−0.0157	0.0071	13	*SDAD1*	Intron	(Fox et al. 2005; Gautier et al. 2011; Iyer et al. 2016); planarian, humans, fish; germ cell development, spermatogenesis and oogenesis

a All QTL positions are based on the lead SNP, rounded to the nearest Mb.

b Additional significant SNPs (*P* ≤ 5e−05) within the same 1 Mb interval as the lead SNP.

**Fig. 2.**
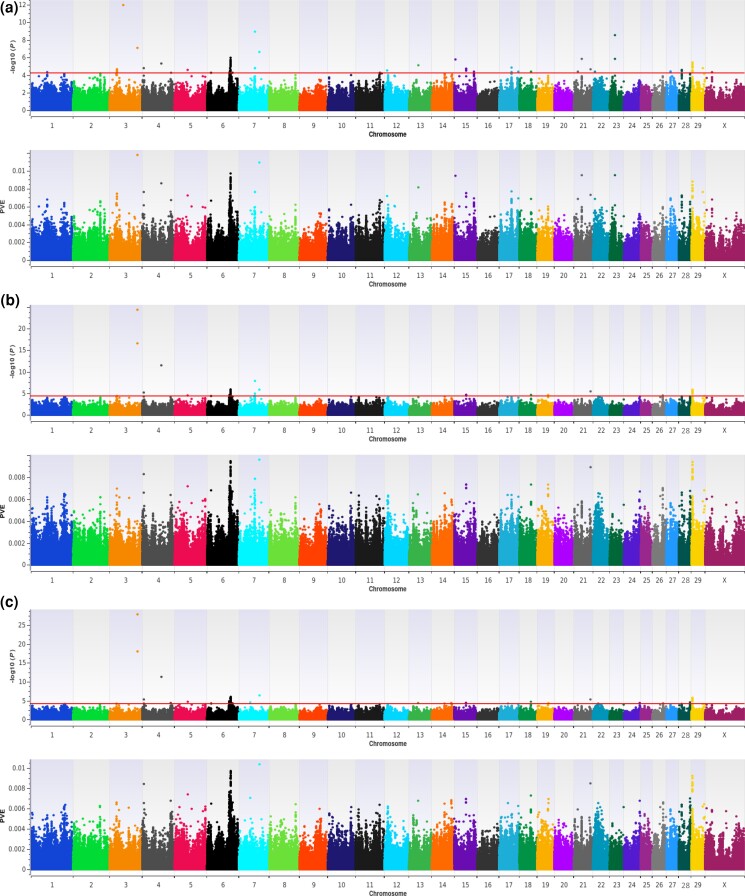
QTL detected for a U.S. Holstein RI by EMMAX (Multi-locus). The dual-panel Manhattan plots depict −log10 *P*-values and the proportion of phenotypic variance explained (PVE) by bovine marker effects on the *y*-axis and the genomic position of all SNPs on the *x*-axis (UMD3.1.1). a) After step 4 of forward inclusion, which was optimal by Multiple Posterior Probability of Association (MPPA); b) After step 15 of backward elimination, which was optimal by Multiple Bonferroni (MBonf) as well as Extended Bayesian Information Criteria (EBIC); c) After step 16 of backward elimination, which was optimal by Modified Bayesian Information Criteria (MBIC). Lead and supporting SNPs for QTL passing a nominal significance threshold for polygenic traits (*P* ≤ 5e − 05; −log_10_*P*-values ≥ 4.30) (Wellcome Trust Case Control Consortium 2007) are represented at or above the red lines for *n* = 2,448 U.S. Holstein cows. A summary of detected QTL, including lead and supporting SNPs passing the nominal significance threshold, is presented in Table 3 and Additional File 2 (File S2) in DRYAD:10.5061/dryad.0gb5mkm04.

A multi-locus EMMAX approach to GWAA which employed both forward inclusion and backward elimination (Segura *et al*. 2012; Vihjálmsson 2012) replicated QTL on BTA6 (84 Mb, 89 Mb) and BTA29 (4 Mb) but also produced evidence for seven additional QTL which met a nominal significance threshold for polygenic traits (*P* ≤ 5e−05; Fig. 2) (Wellcome Trust Case Control Consortium 2007). Six of the seven additional QTL were detected after step 4, which was estimated to be optimal by multiple posterior probability of association (Table 1) and included Holstein RI signals on BTA7 (60 Mb), BTA6 (87 Mb), BTA15 (45 Mb), BTA3 (26 Mb), BTA28 (12 Mb), and BTA27 (15 Mb; Table 3). Thereafter, only one additional Holstein RI QTL was detected for an optimal step (i.e. after step 15, on BTA26 at 40 Mb; Fig. 2), despite some evidence for putative model optimality after steps 6 and 16 (Table 1). All Holstein RI QTL detected after steps 6 and 16 were also detected in other putatively optimal steps (Additional File 2; File S2), thereby demonstrating a high degree of reproducibility among models with different optimality criteria, as would be expected when true associations and/or causal variants exist within the dataset (Segura *et al*. 2012). Multilocus QTL signals detected on BTA7 (60 Mb), BTA6 (87 Mb), BTA15 (45 Mb), BTA3 (26 Mb), BTA28 (12 Mb), BTA27 (15 Mb), and BTA26 (40 Mb) revealed a diverse array of positional candidate genes generally involved in aspects of oocyte maturation and the reproductive longevity pathway (*POU4F3*, *TCERG1*), spermatogenesis and enhanced sperm quality (*CABS1*, *STK33*, *TRNAC-GCA*, and *FAT1*), regulation of T-cell immune responses and early pregnancy pathways (*VTCN1*), increased pregnancy rates (*TRNAC-GCA*, *FAT1*), bovine RD (*TRNAC-GCA*, *ENSBTAG00000042244*, and *TRNAR-ACG*), milk yield (*GRK5*), and the efficacy of ritodrine treatment for arresting preterm labor and birth in humans (*GRK5*) (Table 3; Additional File 2, File S2) (Sica *et al*. 2003; Zanini *et al*. 2003; Maldjian *et al*. 2005; Mujica *et al*. 2005; Zheng *et al*. 2005; Ghazi *et al*. 2009; Cole *et al*. 2011; Guo *et al*. 2011; Aliloo *et al*. 2015; Kaminski *et al*. 2016; Shawki *et al*. 2016; Kong *et al*. 2017; Melo *et al*. 2017; Mikshowsky *et al*. 2017; Hohos *et al*. 2018; Neupane *et al*. 2018; Whittington *et al*. 2018; Chung *et al*. 2020). Interestingly, the positional candidate gene *TRNAC-GCA* was previously implicated in three of the top 20 QTL windows (i.e. on BTA6, BTA11, and BTA29) detected for Nellore heifer fertility, as prioritized by the proportion of variance explained (Melo *et al*. 2017). Herein, we detect a similar signal proximal to the positional candidate gene *TRNAC-GCA*, but on BTA28, and for a U.S. Holstein RI. All multi-locus QTL detected for the U.S. Holstein RI were estimated to have moderate (PVE ≥ 1.0% but <2.0%) or small effects (PVE ≤ 1.0%) on the probability of a pregnancy following AI (Table 3; Additional File 2, File S2; see methods). Genomic inflation factors for each optimal step (after step 4 = 0.9931; after step 6 = 0.9924; after step 15 = 0.9940; and after step 16 = 0.9918) provided no evidence of spurious associations via population substructure.

**Table 3.** Holstein QTL detected by multi-locus EMMAX analysis for a reproductive index (RI).

QTL*^a^* Chr_Mb	EMMAX −log_10_*P*-value	Regression beta	PVE	Supporting SNPs*^c^*	Positional candidate genes	Lead SNP location	Scientific precedence (reference); organism; trait
***7_60* ^*^b^*^**	8.9095	0.0221	0.0076	2	*POU4F3*, *ENSBTAG00000042815*, *TCERG1*	Intergenic	(Zheng et al. 2005; Ghazi et al. 2009); worm, rhesus monkey; reproductive longevity pathway; oocyte differential expression
***6_89***	5.9232	−0.0170	0.0097	33	*SLC4A4*, *LOC782958*, *GC*	Intergenic	(Sahana et al. 2014; Jiang et al. 2019); cattle; milk and protein yields, mastitis, DPR, CCR
***29_04***	5.4183	−0.0160	0.0088	9	*LOC101907665*, *LOC104976210*	Intergenic	NA
***6_87***	4.7291	0.0160	0.0075	2	*LOC104968905*, *ENSBTAG00000027531*, *CABS1*	Intergenic	(Shawki et al. 2016); pigs; function in spermatogenesis and elongation of spermatids
***15_45***	4.7131	0.0643	0.0075	4	*STK33*	Intron	(Mujica et al. 2005; Kong et al. 2017); mouse, human; cell regulation, spermatogenesis, expressed in testis tissue
***3_26***	4.6611	0.0330	0.0074	4	*LOC101904405*, *VTCN1*	3'UTR, Intergenic	(Sica et al. 2003; Whittington *et al*. 2018); mouse, marsupials; regulation of T-cell immune responses, differentially expressed in early pregnancy
***28_12***	4.5456	−0.0238	0.0072	2	*TRNAC-GCA*, *ENSBTAG00000042244*, *TRNAR-ACG*	Intergenic	(Kaminski *et al*. 2016; Melo *et al*. 2017; Cole *et al*. 2011; Neupane *et al*. 2018); cattle; fertility, sperm membrane integrity, heifer rebreeding*^b^*, body depth, bovine respiratory disease
***27_15***	4.3710	0.0135	0.0069	2	*FAT1*	Intron	(Zanini *et al*. 2003; Maldjian *et al*. 2005; Guo *et al*. 2011; Hohos *et al*. 2018); cattle, chickens, pigs, mice; produces high levels of *n*-3 fatty acids, regulation of fatty acid metabolism, enhanced semen quality, increased pregnancy rates and shorter times to pregnancy

a All QTL positions (after step 4) are based on the lead SNP, rounded to the nearest Mb.

b The lead SNP at 7_60 is a cofactor after step 4 (see methods); PVE estimated from the supporting SNPs.

c Additional significant SNPs (*P* ≤ 5e − 05) within the same 1 Mb interval as the lead SNP.

### Genomic prediction with cross validation for a U.S. Holstein RI

To investigate the potential for including the U.S. Holstein RI (Lopes *et al*. 2020; Pinedo *et al*. 2020) in existing or future genomic selection initiatives, we used GBLUP in conjunction with *k*-fold cross validation and random sampling (Taylor 2014; Seabury *et al*. 2020). Briefly, U.S. Holstein data (i.e. genotypes, RI values, and other metadata such as farm and season of calving) were randomly partitioned into *k*-subsamples (*k* = 3), with one subsample selected (i.e. within a discrete fold) to serve as the validation set for genomic prediction. Thereafter, the GBLUP model was fit using the remaining (i.e. *k*-1) Holstein subsamples within that fold (i.e. the training data), until all subsamples were used for both training and prediction (Taylor 2014; Seabury *et al*. 2020). All U.S. Holstein cross validations were run for 10 iterations using random sampling, with each iteration consisting of three folds (*k* = 3), and relevant summary statistics were produced (see Table 4; methods; Additional File 3, File S3). Holstein GBLUP models fit with no fixed-effect covariates (i.e. farm and season) performed best with respect to classical measures such as mean predictive ability and mean genomic prediction accuracy, as previously defined (Bolormaa *et al*. 2013; Mikshowsky *et al*. 2017; Lopes *et al*. 2020) (Table 4). However, when comparatively considering all predictive correlations estimated here, GBLUP models fit with farm and season were observed to perform well with respect to predicting the probability of pregnancy following AI, as evidenced by the mean correlation between the predicted Holstein RI phenotypes and the known Holstein RI phenotypes (Table 4; see methods). This result was not totally unexpected considering how the U.S. Holstein RI was developed (Lopes *et al*. 2020; Pinedo *et al*. 2020) (see methods). Mean predictive abilities and mean genomic prediction accuracies produced here for the U.S. Holstein RI were often similar to those previously produced for a variety of Holstein health traits as well as feed efficiency, carcass, and meat traits in beef cattle, but lower than those estimated for Holstein DPR (Bolormaa *et al*. 2013; Mikshowsky *et al*. 2017; Lopes *et al*. 2020). Nevertheless, the U.S. Holstein RI utilized here directly accounts for a variety of fixed effects and interactions that have previously been shown to influence the probability of a pregnancy following AI, all of which are not directly accounted for in the estimation of DPR (AIPL 2005; Garcia-Ruiz *et al*. 2016; Mikshowsky *et al*. 2017; Kiser *et al*. 2019). Moreover, DPR is known to be lowly heritable, particularly in comparison to the genomic heritability estimates produced for the U.S. Holstein RI in this study (Table 1) (AIPL 2005; Garcia-Ruiz *et al*. 2016; Mikshowsky *et al*. 2017; Kiser *et al*. 2019). Therefore, utilization of the U.S. Holstein RI for genome-enabled predictions may potentially add value to existing or future genomic selection initiatives aimed at improving U.S. dairy cow and heifer fertility.

**Table 4.** Summary of genomic predictions with cross validation (*k* = 3) for a Holstein reproductive index (RI).

Holstein RI *k*-fold subsample	Holstein RI GBLUP model covariates	Mean predictive ability (SD)*^a^*	Mean prediction accuracy (SD)*^b^*	Mean Pearson's (*R*) (SD)*^c^*	Mean *R*-squared (SD)	Mean RMSE (SD)*^d^*	Mean MAE (SD)*^e^*
***k* = 3**	None	0.2301 (0.0127)	0.4557 (0.0251)	0.2288 (0.0124)	0.0521 (0.0061)	0.1169 (0.0004)	0.0970 (0.0002)
***k* = 3**	Farm, Season	0.1692 (0.0130)	0.4119 (0.0317)	0.3656 (0.0059)	0.1330 (0.0049)	0.1118 (0.0003)	0.0906 (0.0001)

a Mean Pearson's product–moment correlation coefficient between the genomically estimated breeding values (GEBVs) and the Holstein RI phenotypes, with standard deviation (SD) (Lopes *et al*. 2020).

b Mean Pearson's product–moment correlation coefficient between the genomically estimated breeding values (GEBVs) and the Holstein RI phenotypes, divided by the square root of the genomic heritability estimate, with standard deviation (SD) (Bolormaa *et al*. 2013; Seabury *et al*. 2020).

c Mean Pearson's product–moment correlation coefficient between the predicted Holstein RI phenotypes and the known Holstein RI phenotypes, with standard deviation (SD) (Seabury *et al*. 2020).

d Root mean square error (RMSE; see methods) (Seabury *et al*. 2020).

e Mean absolute error (MAE; see methods) (Seabury *et al*. 2020).

### Conclusions

Herein, we use a recently developed measure of U.S. Holstein fertility (RI) to conduct two GWAAs and subsequently detect a major QTL on BTA6 for DPR and CCR, but also produce evidence for moderate genomic heritability estimates as well as 12 additional QTL affecting the probability of a pregnancy following AI. Positional candidate genes identified for the detected QTL include a variety of male and female fertility loci, known milk yield QTL, loci generally associated with the cell cycle, and one QTL interval previously associated with bovine RD. Genomic prediction with three-fold cross validation produced evidence in support of potentially utilizing the U.S. Holstein RI for improving fertility in U.S. dairy cows and heifers. Moreover, our ability to capture a major-effect DPR and CCR QTL combined with moderate heritability estimates strongly suggests that the U.S. Holstein RI might be useful in genomic selection initiatives. Collectively, the results of this study demonstrate that new measures of Holstein fertility can lead to changes in knowledge and perhaps changes in action with respect to genetic improvement of U.S. dairy cattle.

## Supplementary Material

jkad043_Supplementary_Data

## Data Availability

Accession codes are as follows: all data and additional files (DRYAD: 10.5061/dryad.0gb5mkm04). Supplemental material available at G3 online.
