# Supplementary material for: Genome-wide association and genomic prediction for a reproductive index summarizing fertility outcomes in U.S. Holsteins
Source: G3 (Bethesda). 2023 Feb 27;13(9):jkad043. doi: 10.1093/g3journal/jkad043 (PMC10468724; doi:10.1093/g3journal/jkad043)
Supplement: jkad043_Supplementary_Data [file jkad043_supplementary_data.docx]

**Supplemental File 1: Genome-Wide Association and Genomic Prediction for a Reproductive Index Summarizing Fertility Outcomes in U.S. Holsteins**


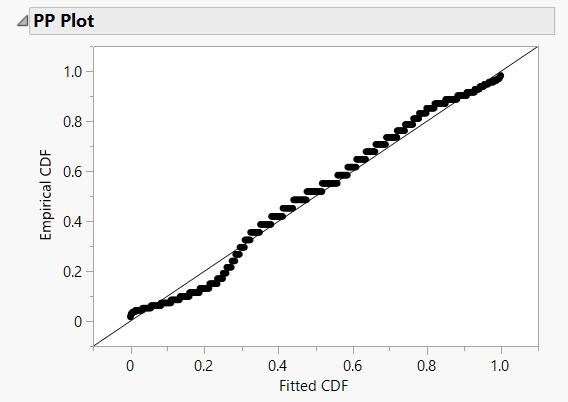


**Figure S1**: PP-Plot of the RI Empirical CDF and the RI Fitted (Normal) CDF.

**Supplemental References for Table 2 and Table 3**

[1] Jiang J, Ma L, Prakapenka D, VanRaden PM, Cole JB, Da Y. A large-scale genome-wide association study in U.S. Holstein cattle. Front Genet. 2019;14 May, https://doi.org/10.3389/fgene.2019.00412.

[2] Sahana G, Guldbrandtsen B, Thomsen B, Holm LE, Panitz F, Brøndum RF, et al. Genome-wide association study using high-density single nucleotide polymorphism arrays and whole-genome sequences for clinical mastitis traits in dairy cattle. J Dairy Sci. 2014;97:7258–75.

[3] Killeen AP, Morris DG, Kenny DA, Mullen MP, Diskin MG, Waters SM. Global gene expression in endometrium of high and low fertility heifers during the mid-luteal phase of the estrous cycle. BMC Genomics. 2014;15

[4] Saitoh H, Tomkiel J, Cooke CA, Ratrie H, Maurer M, Rothfield NF, et al. CENP-C, an autoantigen in scleroderma, is a component of the human inner kinetochore plate. Cell. 1992;70:115–25.

[5] Przewloka MR, Venkei Z, Bolanos-Garcia VM, Debski J, Dadlez M, Glover DM. CENP-C is a structural platform for kinetochore assembly. Curr Biol. 2011;21:399–405.

[6] Kwend L, Collins CM, Dattoli AA, Dunleavy EM. Nucleolar activity and CENP-C regulate CENP-A and CAL1 availability for centromere assembly in meiosis. Dev. 2016;143:1400–12

[7] Gautier A, Le Gac F, Lareyre JJ. The gsdf gene locus harbors evolutionary conserved and clustered genes preferentially expressed in fish previtellogenic oocytes. Gene. 2011;472:7–17.

[8] Fox M, Urano J, Reijo Pera RA. Identification and characterization of RNA sequences to which human PUMILIO-2 (PUM2) and deleted in Azoospermia-like (DAZL) bind. Genomics. 2005;85:92–105.

[9] Iyer H, Issigonis M, Sharma PP, Extavour CG, Newmark PA. A premeiotic function for boule in the planarian Schmidtea mediterranea. PNAS. 2016;113.

[10] Zheng P, Patel B, McMenamin M, Moran E, Paprocki AM, Kihara M, et al. Effects of Follicle Size and Oocyte Maturation Conditions on Maternal Messenger RNA Regulation and Gene Expression in Rhesus Monkey Oocytes and Embryos1. Biol Reprod. 2005;72:890–7.

[11] Ghazi A, Henis-Korenblit S, Kenyon C. A transcription elongation factor that links signals from the reproductive system to lifespan extension in Caenorhabditis elegans. PLoS Genet. 2009;5.

[12] Shawki HH, Kigoshi T, Katoh Y, Matsuda M, Ugboma CM, Takahashi S, et al. Identification, localization, and functional analysis of the homologues of mouse CABS1 protein in porcine testis. Exp Anim. 2016;65:253–65.

[13] Mujica AO, Brauksiepe B, Saaler-Reinhardt S, Reuss S, Schmidt ER. Differential expression pattern of the novel serine/threonine kinase, STK33, in mice and men. In: FEBS Journal. 2005. p. 4884–98.

[14] Kong F, Kong X, Du Y, Chen Y, Deng X, Zhu J, et al. STK33 Promotes Growth and Progression of Pancreatic Cancer as a Critical Downstream Mediator of HIF1α. Cancer Res. 2017;77:6851–62. doi:10.1158/0008-5472.CAN-17-0067.

[15] Sica GL, Choi IH, Zhu G, Tamada K, Wang SD, Tamura H, et al. B7-H4, a Molecule of the B7 Family, Negatively Regulates T Cell Immunity. Immunity. 2003;18:849–61.

[16] Whittington CM, O’Meally D, Laird MK, Belov K, Thompson MB, McAllan BM. Transcriptomic changes in the pre-implantation uterus highlight histotrophic nutrition of the developing marsupial embryo. Sci Rep. 2018;8.

[17] Kaminski S, Hering DM, Olenski K, Lecewicz M, Kordan W. Genome-wide association study for sperm membrane integrity in frozen-thawed semen of Holstein-Friesian bulls. Anim Reprod Sci. 2016;170:135–140.

[18] Melo TP, de Camargo GMF, de Albuquerque LG, Carvalheiro R. Genome-wide association study provides strong evidence of genes affecting the reproductive performance of Nellore beef cows. PLoS One. 2017;12(5):e0178551.

[19] Cole JB, Wiggans GR, Ma L, Sonstegard TS, Lawlor TJ Jr, Crooker BA, et al. Genome-wide association analysis of thirty one production, health, reproduction and body conformation traits in contemporary U.S. Holstein cows. BMC Genomics. 2011;12:408. https://doi.org/10.1186/1471-2164-12-408.

[20] Neupane M, Kiser JN, Bovine Respiratory Disease Complex Coordinated Agricultural Project Research Team, Neibergs HL. Gene set enrichment analysis of SNP data in dairy and beef cattle with bovine respiratory disease. Anim Genet. 2018;49:527-538.

[21] Hohos NM, Cho KJ, Swindle DC, Allshouse AA, Rudolph MC, Skaznik-Wikiel ME. Fat-1 Transgene Is Associated With Improved Reproductive Outcomes. Endocrinology. 2018;159(12):3981-3992.

[22] Guo T, Liu XF, Ding XB, Yang FF, Nie YW, An YJ, et al. Fat-1 transgenic cattle as a model to study the function ofω-3 fatty acids. Lipids Health Dis. 2011;10.

[23] Maldjian A, Pizzi F, Gliozzi T, Cerolini S, Penny P, Noble R. Changes in sperm quality and lipid composition during cryopreservation of boar semen. Theriogenology. 2005;63:411-421.

[24] Zanini SF, Torres CA, Bragagnolo N, Turatti JM, Silva MG, Zanini MS. Evaluation of the ratio of omega-6: omega-3 fatty acids and vitamin E levels in the diet on the reproductive performance of cockerels. Arch Tierernahr. 2003;57:429-442.
